# Mutations in the tail domain of *MYH3* contributes to atrial septal defect

**DOI:** 10.1371/journal.pone.0230982

**Published:** 2020-04-21

**Authors:** Sathiya Maran, Robson Ee, Siti Aisyah Faten, Choi Sy Bing, Kooi Yeong Khaw, Swee-Hua Erin Lim, Kok-Song Lai, Wan Pauzi Wan Ibrahim, Mohd Rizal Mohd Zain, Kok Gan Chan, Siew Hua Gan, Huay Lin Tan

**Affiliations:** 1 School of Pharmacy, Monash University, Sunway, Selangor, Malaysia; 2 Human Genome Centre, Universiti Sains Malaysia, Kubang Kerian, Kelantan, Malaysia; 3 Division of Genetics and Molecular Biology, Faculty of Science, Institute of Biological Sciences, University of Malaya, Kuala Lumpur, Malaysia; 4 School of Data Sciences, Perdana University, Selangor, Malaysia; 5 Health Sciences Division, Abu Dhabi Women's College, Higher Colleges of Technology, Abu Dhabi, United Arab Emirates; 6 Faculty of Medicine and Health Sciences, Universiti Sultan Zainal Abidin, Kuala Terengganu, Terengganu, Malaysia; 7 Department of Paediatrics, School of Medical Sciences, Universiti Sains Malaysia, Kubang Kerian, Kelantan, Malaysia; 8 International Genome Centre, Jiangsu University, Zhenjiang, China; Jagiellonian University Medical College, POLAND

## Abstract

Atrial septal defect (ASD) is one of the most common congenital heart defects diagnosed in children. Sarcomeric genes has been attributed to ASD and knockdown of *MYH3* functionally homologues gene in chick models indicated abnormal atrial septal development. Here, we report for the first time, a case-control study investigating the role of *MYH3* among non-syndromic ASD patients in contributing to septal development. Four amplicons which will amplifies the 40 kb *MYH3* were designed and amplified using long range-PCR. The amplicons were then sequenced using indexed paired-end libraries on the MiSeq platform. The STREGA guidelines were applied for planning and reporting. The non-synonymous c. 3574G>A (p.Ala1192Thr) [p = 0.001, OR = 2.30 (1.36–3.87)] located within the tail domain indicated a highly conserved protein region. The mutant model of c. 3574G>A (p.Ala1192Thr) showed high root mean square deviation (RMSD) values compared to the wild model. To our knowledge, this is the first study to provide compelling evidence on the pathogenesis of *MYH3* variants towards ASD hence, suggesting the crucial role of non-synonymous variants in the tail domain of *MYH3* towards atrial septal development. It is hoped that this gene can be used as panel for diagnosis of ASD in future.

## Introduction

Atrial septal defect (ASD) is one of the most common forms of congenital heart defects (CHD), with an estimated incidence of an approximately 100 per 100 000 live births [1] and accounting for 10–15% of CHD worldwide [2–4]. Based on the location relative to the fosa ovalis, ASD refers to a communication between the right and left atria, anatomically classified into the deficient of atrial septum structure [5]. ASD is categorized into 1) ostium secundum 2) ostium primum 3) sinus venosus and [6]. An uncorrected ASD can cause a pulmonary over-circulation thus generating a right heart volume overload. The increased pulmonary resistance and pressure due to histopathological pulmonary arteriolar changes can contribute to a reduced functional capacity, leading to cyanosis, arrhythmia, stroke and a premature death [7]. Factors such as environment, maternal factors, specialised medications during pregnancy and genetic variants may disturb the regulatory factors of heart, leading to aberrant structure and function of heart [8].

The incidence of CHD among Asians has been reported to be 50% higher compared to the Caucasians and Blacks, especially for severe and complex CHD that have a high infant mortality[9]. In Asian countries, the incidence of ASD is high in Malaysia (9.6%) [10], Korea (44.2%) [11] and Thailand (43%) [12]. Studies have described that approximately 5% of children with serious CHD dies without undergoing any procedures [13]. The influence of genetic factors in contributing to a high incidence of CHD among Asians has not been clearly described despite reports indicating a potentially higher exposure due to genetic factors [14]. This underpins the importance of investigating the genetic causative factor of ASD among Asian, especially in Malaysia.

The molecular genetics of ASD have been elucidated with mutations in transcription factor genes; *Nkx2*.*5*, *Tbx5* and *Gata4* and structural proteins; *MYH6*, *MYH7* and α-cardiac action [15]. Although these mutations have provided important insights into cardiac morphogenesis and ASD formation, the causative gene remains unknown in many families and individuals [16]. A study by Ruthland and colleagues (2011)on chick model showed that knockdown of embryonic myosin heavy chain (eMYH) causes abnormal atrial septal development.According to the study, the atrial septum either failed to form or only a small outgrowth of the dorsocranial wall was observed, or a small septum is formed in comparison with controls. These lead to an interest in comprehending the possible homologous relationships between the human and the chick’ MYH clusters. Thus, an expression profile of human skeletal myosin heavy chain genes carried out suggested that the human myosin heavy chain 3 (*MYH3*) is the functional homolog of the chick’ eMYH gene [17].

*MYH3* (OMIM#160720) is a sarcomeric muscle gene mapped to chromosome 17, consisting of 40 coding exons spanning to a total of 40 kb [18]. The *MYH3* belongs to the myosin II family, which is composed of globular motor domain, a short neck and hinge region connected to a long coiled-coil rod domain [19]. *MYH3* which is expressed during fetal development and muscle regeneration has previously been associated with Freeman-Sheldon and Sheldon-Hall syndromes [20], although defects on the heart is yet to be described [18].

Studies have reported the contributing factors of *MYH3* towards distal arthrogryposis type I, 2A (Freeman-Sheldon syndrome) and 2B (Sheldon-Hall syndrome), contractures, pterygia, and variable skeletal fusions syndrome 1A (OMIM 178110) and 1B (OMIM 618469). However, to the best of our knowledge, the association of MYH3 towards heart abnormalities in humans has not been reported. Therefore, this study for the first time reports genetic association of *MYH3* towards atrial septal formation among non-syndromic ASD patients.

## Materials and methods

### Study population and sample collection

This was a case-control study approved by the Research and Ethics Committee, School of Medical Sciences, USM Health Campus (USMKK/PPP/JEPeM [252.3(13)]) and Ministry of Health Malaysia (MOH) (KKM/NIHSEC/BOO-2/2/2/P13-147) which complies with the Declaration of Helsinki. Written informed consents to participate in this study were obtained from either the parents of patients below 18 years old or directly from the patients who are 18 years old and above. Additionally, all patients and/or parent’ of patients has signed written informed consents to allow publication of their medical and/or genetic information.

A total of 69 non-syndromic ASD patients (cases) confirmed with ASD were recruited within almost one year. Baseline assessment of individual and family histories, review of the medical records and two-dimensional echocardiography with colour flow Doppler by an experienced paediatric cardiologist was recorded. For each case recruited, a gender-ethnicity matched control was recruited, this is to avoid analysis stratification. Echocardiography was also carried-out on the controls to eliminate potential bias. The recruitment of patients and controls cohort was conducted at Echocardiography Unit, Hospital Universiti Sains Malaysia (HUSM), Kubang Kerian, Kelantan, which is the main tertiary cardiac referral centre in the northeast region of Peninsular Malaysia. Following signing of written informed consents, peripheral blood (3 ml) was collected in EDTA tubes and stored at 4°C until further analysis.

### Long-range PCR development and amplification

Genomic DNA of the recruited samples were extracted from peripheral blood from patients (n = 44) using a commercially available kit GeneAll^®^ Exgene^TM^ Blood SV mini, GeneAll, Korea, at the Human Genome Center, Universiti Sains Malaysia (HGC-USM). For patients who refused to give their blood (n = 25), saliva samples were collected instead using PSP® SalivaGene DNA kit, STRATEC. Findings from a high-throughput genotyping study reported that DNA isolates from blood draws and DNA isolates form saliva showed no comparable differences in term of results [21]. The concentration and purity of the extracted DNA was measured using NanoQuant (Tecan, USA).

*MYH3* sequence from Ensembl Genome Browser (ID: ENSG00000109063, GRCh38) was used to design the LR-PCR primers. The entire promoter, 5’ and 3’ *MYH3* untranslated and coding regions were amplified in four distinct LR-PCR reactions. LR-PCR was performed using a Max Taq Polymerase (Vivantis, USA), according to the manufacturer's protocol at the HGC-USM.

DNA thermal cycling was performed using a SureCycler 8800 (Agilent Technologies, UK); 94°C for 2 min, 94°C 12 sec, annealing temperature for 30 secs, 68°C for 10 mins, 68°C for 7 mins for a total of 35 cycles. The size of the amplified PCR products was determined by gel electrophoresis using SYBR® Green I nucleic acid gel stain (Life Technologies, USA) under ultraviolet light. The amplified products were then purified by using an Illustra Exo-ProStar (GE Healthcare, UK). Subsequently, the LR-PCR fragments were quantified using Qubit® 1.0 Fluorometer (Invitrogen, USA) with Qubit dsDNA BR Assay Kits (Invitrogen, USA) and were pooled together at equal molar ratios.

### NGS library preparation and sequencing

For each sample, 5 μl (1 ng in total) at 0.2 ng/ul of pooled LR-PCR products was used to generate indexed paired-end libraries with Nextera XT DNA Sample Preparation Kit (Illumina, San Diego, CA) according to the manufacturer's protocol. A fragment length of the libraries was ascertained using the High Sensitivity DNA kit (Agilent Technologies, Santa Clara, CA) on the 2100 Bioanalyzer (Agilent Technologies, Santa Clara, CA). Normalized libraries were subjected to a 300-cycle sequencing run with MiSeq Reagent Nano Kit v2 (Illumina, San Diego, CA) on the MiSeq (Illumina, San Diego, CA), were carried out the Genetic Laboratory, faculty of Science, University of Malaya.

### Data enrichment and variant calling

The sequencing data was evaluated with FastQC (version 1.0, Babraham Bioinformatics, UK) for quality control on the raw data. Subsequently, the reads were aligned to a reference genome (hg19) by BWA-MEM (version 1.0) algorithm based on the default settings. The SNPs were filtered by a set of filters. A variant Studio v2.1 software (Illumina, San Diego, CA) was used to identify and annotate exonic and intronic variants and to determine if the variants have been reported in public databases. An integrative Genomics Viewer (IGV version v1.0.0) was used to examine the read counts of the target amplicons and confirm the detected variants. All variants identified by NGS were resequenced with Sanger sequencing.

### Genotyping in controls cohort

The identified variants were genotyped in the controls cohort by made-to-order TaqMan SNP Genotyping Assay (ThermoFisher, USA). Variants that are not readily-available were custom designed and genotyped using the TaqMan SNP Genotyping Assay (ThermoFisher, USA). Variants that were not readily available as pre-designed Made-to-order TaqMan® SNP Genotyping Assay and cannot be custom designed were then genotyped using Sanger sequencing method.

### Statistical and bioinformatics analyses

The study protocol and reporting were developed according to the STREGA guidelines for case-control studies [22]. The allelic and genotypic frequencies of the patient and control groups were determined using a Fisher’s exact test with odds ratio and 95% confidence interval (CI). A *x*^2^ *P*-value of < 0.05 was considered as statistically significant. Association was evaluated for every variant and False Discovery Rate and Bonferroni adjustments were used for multiple-testing corrections. Haploview version 4.2 (Broad Institute of MIT and Harvard, USA) was used for analysis of linkage disequilibrium (LD) and haplotype block [23]. LD blocks were defined according to the haplotype block definition of Gabriel and colleagues [24]. The method defines pairs to be in strong LD if the one-sided upper 95% confidence bound on *D*′ is >0.98 and the lower bound is above 0.7.

*In-silico* analysis of pathogenic potential of identified non-synonymous variants were predicted using Sorting Intolerant From Tolerant (SIFT) and Polymorphism Phenotyping v2 (PolyPhen-2). SIFT scores which range between 0 and 1 as well as scores below 0.05 suggest that the amino acid change is not tolerated whereas, PolyPhen-2 scores >0.85 are interpreted as “probably damaging” and scores 0.15–0.85 as “possibly damaging”. The evolutionary conservation of the identified non-synonymous variants was conducted using a Clustal Omega programme (EMBL-EBI, European Bioinformatics Institute). The splicing effects were predicted by the Human Splicing Finder (HSF) version 3.0 (http://www.umd.be/HSF/) and ESEfinder 3.0 (http://rulai.cshl.edu/).

### Modelling of the mutant protein structure

Prior to the template selection, both wild and mutant types of complete sequences of human myosin *MYH3* were aligned against the sequences from Protein Data Bank (PDB), (www.rcsb.org/pdb). Our results showed that protein structures with the PDB ID: 5TBY and 5WJ7 with 72% and 83% sequence identity, respectively are suitable templates for homology modelling. Multiple templates approach homology modelling was adopted in this study. Both the selected templates indicated to belong to the family protein from *Homo sapiens* myosin 7, which is closely related to the modeled sequences.

A total of 20 models for wild and mutant type sequence were built using Modeller 9v20 (www.salilab.org/modeller) and the best models with the lowest discrete optimize potential energy (DOPE) scoring was selected for each wild and mutant types. The structural differences between the mutant against the wild type built model was calculated using a root mean square deviation (RMSD) method.

### Nomenclature and classification for detected variants

All sequence variants are named according to the nomenclature guidelines published by the Human Genome Variation Society (HGVS) [25]. Nucleotide and amino acid numbering were based on GenBank reference sequences NM_002470.3 and NP_002461.2 respectively.

## Results

In the present study, a total of 69 non-syndromic ASD patients were recruited. However, only 51 samples were further proceeded as 18 samples were excluded due to low genomic DNA concentration (<50 ng/ul) and low sequencing libraries of <10 nM.

The entire 40 kb of *MYH3* was successfully sequenced in 51 non-syndromic ASD patients using LRPCR-NGS approach. The validity of the results was confirmed with Sanger sequencing.

### Demographic and clinical data

Although a total of 69 non-syndromic ASD patients where recruited (blood sample, n = 44 and saliva sample, n = 25), only 51 samples were further proceeded for sequencing. The demographic data of these 51 samples were; aged between 3–62 years old with mean age of 4.72 ± 1.77 years. Based on ethnicity, 47 (92.16%) were Malays, two (3.92%) were Chinese and two (3.92%) were Siamese. In terms of gender, 4 (28.6%) were male and 47 (71.4%) were female patients. The cardiac phenotypes of the recruited patients were categorized into four groups 1) ASD secundum (82.2%) 2) ASD primum (6.5%) and 3) fenestrated ASD (11.3%). The recruited controls cohort consisting of 73 healthy individuals were matched for age, ethnicity and gender with the cases. The age of controls ranged from 3–62 years old with mean age of 4.72 ± 1.77 years. In term of ethnicity and gender; 69 Malays, two Chinese and two Siamese, with 59 of them being females and 14 males.

### Coverage and sequencing depth of targets of LRPCR-NGS

Four pairs of LR-PCR primers were designed to amplify the entire 40 kb of *MYH3*. These primers were optimized at annealing temperatures of 63.1°C, 62.4°C, 64.7°C and 64.7°C respectively, employing LR-PCR cycling method within a short period of time. Targeted sequencing using Illumina MiSeq paired-end sequence resulted in 11,947,785 reads (98.05%) of which 99.8% may be aligned with the human genome (S1 Fig).

This provided 99.8% coverage of the sequence at a minimum sequencing depth of 6×. The run had a cluster density of 615 +/- 6 K/mm^**2**^ with approximately 92.63 +/- 2.05% of the clusters passing the QC filter. The observed read depth in all the samples was > 100 X per base with Q ≥ 30 (99.9% base call accuracy).

### *MYH3* variants analysis

**Fig 1:**
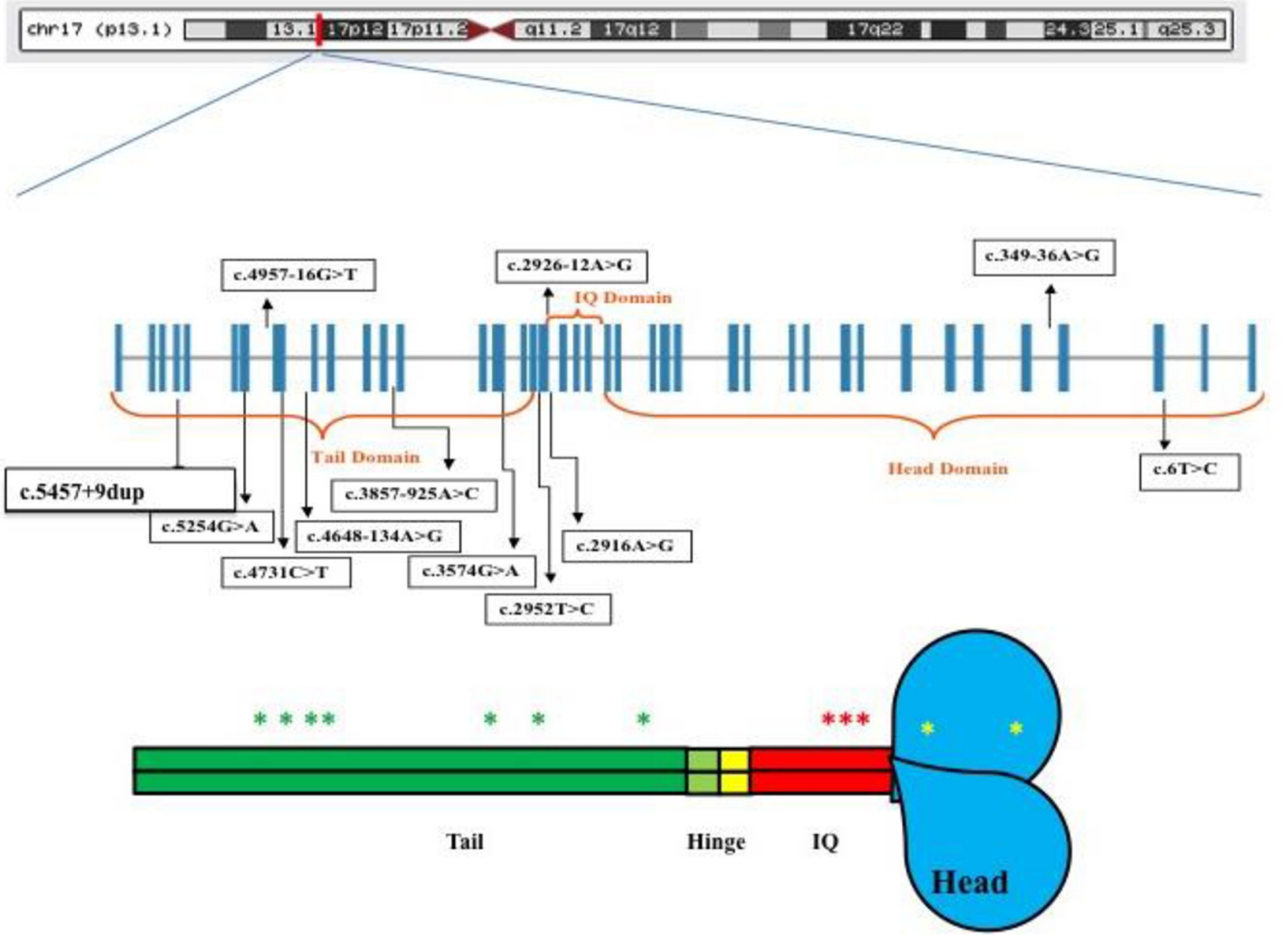
Genomic model of *MYH3* and diagrammatic illustration of the spectrum of variants. (A) Position of *MYH3* at Chromosome 17. (B) Boxes represent exons and adjoining lines represent introns. (C) Schematic representation of *MYH3* domains with the approximate location of *MYH3* variants indicated with coloured asterisk mark.

Variants with minor allele frequency (MAF) <5% and phred scale of <30 were excluded, resulting in 11 variants and one indel. Fig 1B and 1C show the diagrammatic position of the *MYH3* variants (Fig 1) detected while Table 1 summarizes the characteristic of the identified variants. To evaluate the performance and validity of the LRPCR-NGS, resequencing of the identified variants was carried out using a Sanger sequencing.

**Table 1:** *MYH3* variants detected using LRPCR-NGS approach.

Variants	HGVS	rs ID	Location	Amico Acid Change	Type of variant	PolyPhen-2	SIFT	Number of patients	Type of ASD
									(n = number of patients)
c.6T>C	NC_000017.11:g.10655059A>G	rs17817203	Exonic	-	Synonymous	-	-	2	• Fenestrated ASD (n = 1)
									• ASD secundum (n = 1)
c.2952T>C	NC_000017.11:g.10639448A>G	rs2285474	Exonic	-	Synonymous	-	-	22	• ASD secundum (n = 19)
									• ASD primum (n = 2)
									• Fenestrated ASD (n = 1)
c.4648-134A>G	NC_000017.11:g.10632918T>C	rs1981514	Intronic	-	-	-	-	28	• ASD secundum (n = 23)
									• ASD primum (n = 3)
									• Fenestrated ASD (n = 2)
c.5254G>A	NC_000017.11:g.10631643C>A	rs34393601	Exonic	p.Ala1752Thr	Missense	0.144	0.01	2	• ASD secundum (n = 1)
						(Benign)	(Deleterious)		• Fenestrated ASD (n = 1)
c.4957-16G>T	ENST00000583535.6:c.4957-16G>T	rs2239936	Intronic	-	-	-	-	36	• ASD secundum (n = 30)
									• ASD primum (n = 3)
									• Fenestrated ASD (n = 3)
c.2916A>G	ENST00000583535.6:c.2916A>G	rs2285472	Exonic	-	Synonymous	-	-	25	• ASD secundum (n = 22)
									• ASD primum (n = 2)
									• Fenestrated ASD (n = 1)
c.2926-12A>G	ENST00000583535.6:c.2926-12A>G	rs2285473	Intronic	-	-	-	-	22	• ASD secundum (n = 20)
									• ASD primum (n = 2)
c.3574G>A	ENST00000583535.6:c.3574G>A	rs2285477	Exonic	p.Ala1192Thr	Missense	0.003	0.44	27	• ASD secundum (n = 21)
						(Benign)	(Tolerated)		• ASD primum (n = 3)
									• Fenestrated ASD (n = 3)
c.4731C>T	ENST00000583535.6:c.4731C>T	rs2285479	Exonic	-	Synonymous	-	-	32	• ASD secundum (n = 30)
									• ASD primum (n = 1)
									• Fenestrated ASD (n = 1)
c.3857-925A>C	ENST00000583535.6:c.3857-925A>C	rs55980976	Intronic	-	-	-	-	31	• ASD secundum (n = 29)
									• ASD primum (n = 2)
c.5457+9dup	NC_000017.11:g.10630279dup	rs397750512 INDEL	Intronic	-	Insertion	-	-	33	• ASD secundum (n = 30)
									• ASD primum (n = 2)
									• Fenestrated ASD (n = 1)
c.349-36A>G	ENST00000583535.6:c.349-36A>G	rs2285467	Exonic	-	Synonymous	-	-	12	• ASD secundum (n = 12)

### Statistical and bioinformatics analysis

**Fig 2:**
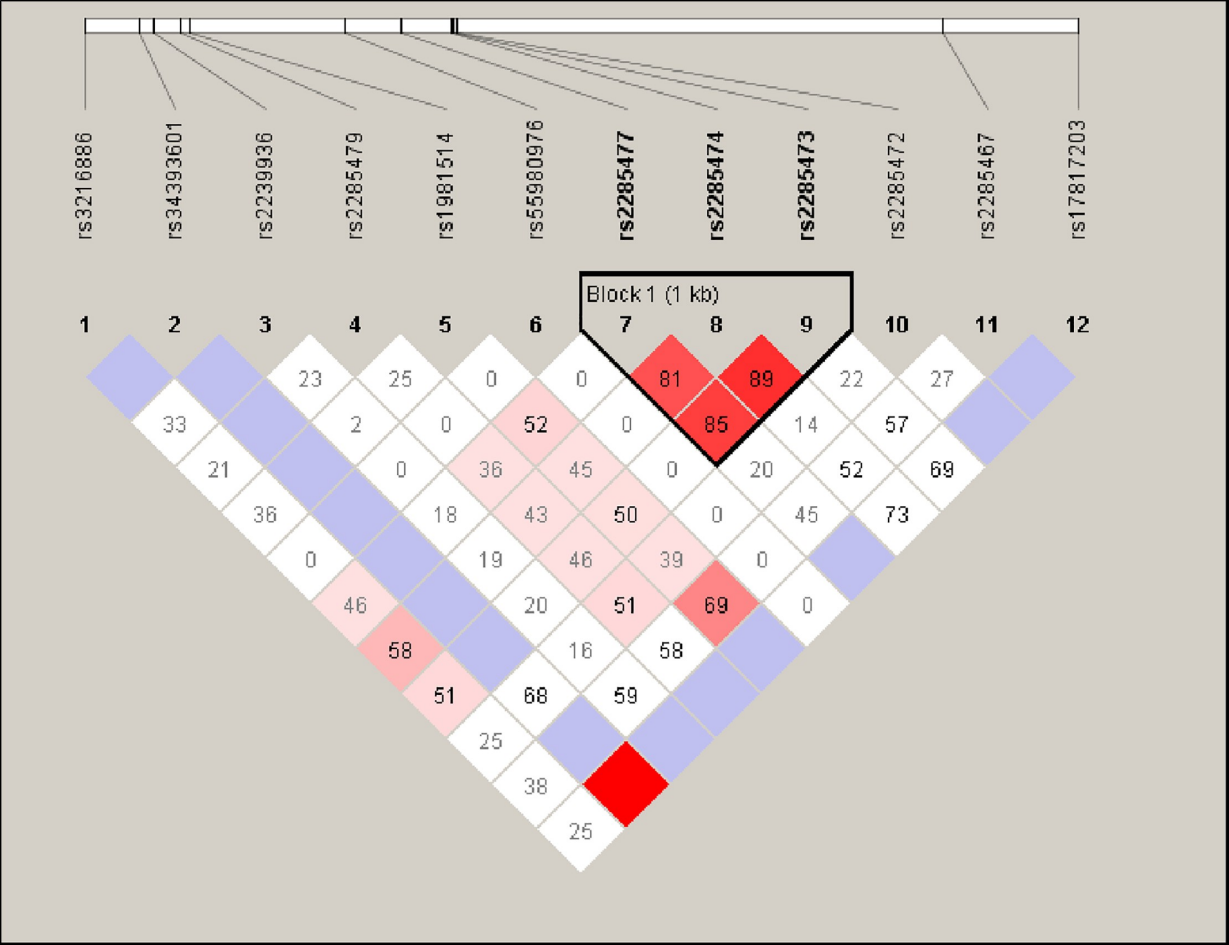
Haploview generated graphical representation of LD in *MYH3* variants identified among ASD patients. Twelve SNPs spanning from position 17:10655059 to 17:10631643 were grouped into one block consisting of rs2285477 (c.3574G>A), rs2285474 (c.2952T>C) and rs2285473 (c.2926-12A>G) according to the LD structure. Length of the block is provided in kilobases (kb), and pairwise LD (D′) is given for each SNP combination (showed in box). The color code in the haploview plot follows the standard color scheme for haploview: white, |D′|<1, LOD<2; shades of pink, |D′|<1, LOD≥2; blue, | D′| = 1, LOD<2; red, |D′| = 1, LOD≥2.

Fisher exact *x*^2^ analysis showed c.4648-134A>G (p = 0.007, OR = 2.05 [CI: 1.18–3.57]), c.2952T>C (p = 0.0003, OR = 2.62 [CI: 1.54–4.45]), c.4957-16G>T (p = 0.044, OR = 0.62 [CI:0.371.03]), c.2916A>G (p = 0.0003, OR = 2.55 [CI: 1.50–4.32]), c.2926-12A>G (p = 0.00006, OR = 2.90 [CI: 1.69–4099]), c.3574G>A (p = 0.001, OR = 2.30 [CI: 1.36–3.87]), c. 349–36 A>G (p = 0.033, OR = 0.37[CI: 0.14–0.97]) and c.3857-925A>C (p = 0.000, OR = 0.00 [CI:0. 00–0.06]) to be significantly correlated with ASD (Table 2). These variants were further subjected to haplotype analysis. The LD block of rs2285477 (c.3574G>A), rs2285474 (c.2952T>C) and rs2285473 (c.2926-12A>G) showed a strong correlation with ASD (p-value; GTA: 0.0003, ACG: 2.878e-5) (Fig 2), haplotypes: GTA, ACG and ATG also showed statistical significant (p<0.05) when compared to the control group.

**Table 2:** Genotype and allelic frequencies distribution of the variants in a cohort of ASD patients and healthy controls.

Variant	Variables	ASD patients (n = 51)	Control population (n = 73)	p-value	Odds Ratio (95% CI)
c.6T>C	Genotype				
	T/T	49	70		
	C/C	-	3		
	T/C	2	-	0.288	2.14 (0.42–10.83)
	Allele Frequency				
	T	0.980	0.958		
	C	0.019	0.042		
c.4648-134A>G	Genotype				
	T/T	23	11		
	C/C	8	13		
	T/C	20	32	0.011	1.97 (1.13–3.41)
	Allele Frequency				
	T	0.647	0.369		
	C	0.353	0.397		
c.2952T>C	Genotype				
	T/T	27	15		
	C/C	7	17		
	T/C	17	41	P = 0.0002	2.62 (1.54–4.45)
	Allele Frequency				
	T	0.696	0.486		
	C	0.303	0.514		
c.5254G>A	Genotype				
	G/G	49	73		
	A/A	-	0		
	G/A	2	-		
	Allele Frequency			0.172	0.00 (0.00–0.00
	G	0.980	1.000		
	A	0.019	0		
c.4957-16G>T	Genotype				
	G/G	16	28		
	T/T	14	6		
	G/T	21	38	0.044	0.62 (0.37–1.03)
	Allele Frequency				
	G	0.519	0.644		
	T	0.480	0.342		
c.2916A>G	Genotype				
	A/A	28	15		
	G/G	4	18		
	A/G	19	38	p = 0.0003	2.55 (1.50–4.32)
	Allele Frequency				
	A	0.735	0.466		
	G	0.265	0.507		
c.2926-12A>G	Genotype				
	A/A	29	15		
	G/G	3	17		
	A/G	19	40	p = 00006	2.90 (1.69–4.99)
	Allele Frequency				
	A	0.754	0.479		
	G	0.245	0.507		
c.3574G>A	Genotype				
	G/G	27	14		
	A/A	4	16		
	G/A	20	42	0.001	2.30 (1.36–3.87)
	Allele Frequency				
	G	0.725	0.479		
	A	0.274	0.507		
c.4731C>T	Genotype				
	C/C	17	12		
	T/T	10	14		
	C/T	24	37	0.164	1.34 (0.79–2.26)
	Allele Frequency				
	C	0.568	0.418		
	T	0.431	0.445		
c.3857-925A>C	Genotype				
	AA	21	73		
	CC	9	0		
	AC	21	0	p = 0000	0.00 (0.00–0.06)
	Allele Frequency				
	A	0.617	1.000		
	C	0.382	0.000		
c.5457+9dup	Genotype				
	G/GA	38	7	p≤0.050*	0.01
	GA/GA	13	66		(0.01–0.09)
c.349-36A>G	Genotype				
	AA	38	60		
	GG	1	-		
	AG	12	7	0.033	0.37 (0.14–0.97)
	Allele Frequency				
	A	0.862	0.869		
	G	0.137	0.048		

* p≤0.050 is considered significant

**Fig 3:**
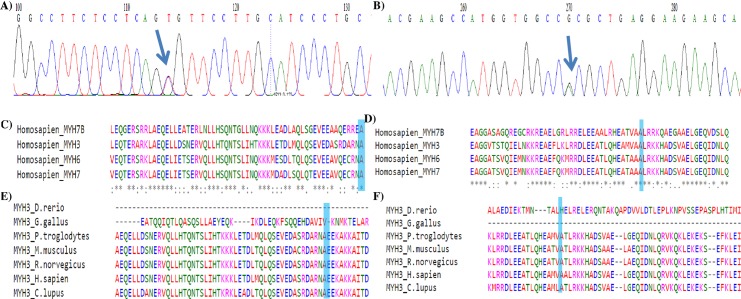
Evolutionary conservation of the non-synonymous (a) c.5254G>A (p.Ala1752Thr, rs34393601) (b) c.3574G>A (p.Ala1192Thr, rs2285477) variant in *MYH3*. The Ensembl accession codes of the paralogous sequences; *MYH7B*: ENSG00000078814, *MYH3*: ENSG00000109063, *MYH6*: ENSG00000197616, *MYH7*, ENSG00000092054; The Ensembl accession codes of the orthologous sequences; zebra fish: C7MYH3.1, chicken: ENSGALG00000002678, chimp: ENSPTRG00000008773, mouse: ENSMUSG00000057003; rat: ENSRNOG00000046276; dog: ENSCAFG0000002309.

The non-synonymous c.5254G>A (p. Ala1752Thr) was predicted to be deleterious by SIFT tool (0.01) whereas PolyPhen-2 predicts it to be benign (0.144). The non-synonymous c.3574G>A (p. Ala1192Thr) was predicted to be tolerated and benign based on SIFT and PolyPhen-2 tool with scores of 0.44 and 0.003 respectively (Table 1). The Ala1751 (p. Ala1752Thr) and Ala1191 (p. Ala1192Thr) residues were identified to be highly conserved in human *MYH7B*, *MYH3*, *MYH6* and *MYH7* whereas in orthologous sequence it is conserved among chimp, mouse, rat, human and dog (Fig 3).

The putative 5’ and 3’ splice sites as well as branch sites were predicted to determine if the identified mutations would disrupt these sites. The results were interpreted by comparing the percentage of consensus values (CV%) between the wild-type and the mutant variant [26]. CV of higher than 80 indicates strong sites, and a negative CV variation percentage between the wild-type and mutant variant indicates that the variant affects splicing mechanism. The c.2916A>G mutation that occurs at late exonic position was predicted to affect the splicing mechanisms. The HSF analysis for c.2916A>G generated consensus values of 80.66 and 51.71 for the wild and mutant types respectively with predicted consensus deviation value of -35.89%. The A to G synonymous substitution (exon 23, 324^th^ nucleotide) affects the SC35 binding motif TGCCACAG, reducing the score from 2.90 to 0.0 (below the default threshold) and creating a sequence containing a potential SRp40 binding motif (TGCCACG score: 2.80) (S2 Fig)

### Modelling of the mutant protein structure

**Fig 4:**
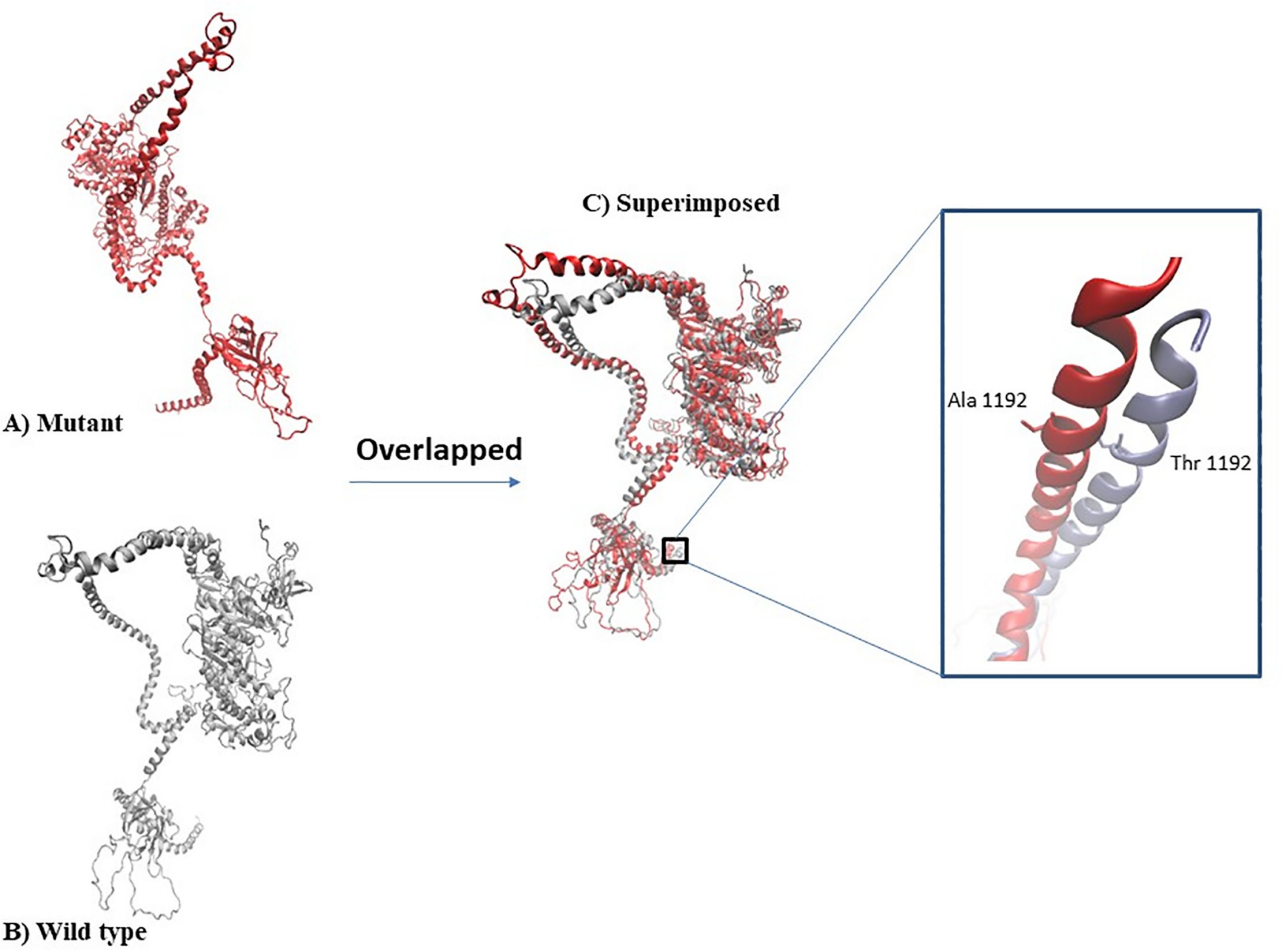
Structural comparison via RMSD analysis (a) Wild type built model (b) Mutant type built model (c) Superimposition of wild and mutant type model with RMSD value of 5.4Å.

Fig 4 shows the 3D structures of the predicted models. The homology modelling of the c. 3574G>A (p.Ala1192Thr) was performed using Visual Molecular Dynamic 1.9.3. (Urbana, IL). The native and wild types models were superimposed, generating RMSD value of 5.4Å. To assess the stereochemical properties of the built model, Ramachandran plot analysis was conducted. Ramachandran plot analysis showed that out of 1121 total residues in both the built models, the wild and mutant type models were observed with 86.5% and 86.1%, residues fall in most favoured region, respectively.

## Discussion

To our knowledge, this is the first study to elucidate the role of *MYH3* towards ASD in non-syndromic patients employing LRPCR-NGS approach. A total of 12 genetic variants were detected of which seven (58%) were located within the tail domain of *MYH3*. The non-synonymous variant c.5254G>A (p. Ala1752Thr) was identified in two female ASD patients having ASD secundum with defect size of 0.5 cm and 0.8 cm respectively but was absent in the 73 unrelated controls. Nevertheless, the non-synonymous c.3574G>A (p. Ala1192Thr) variant was identified in six patients and was associated with ASD [p = 0.001 (OR = 2.30 [1.36–3.87]). Evolutionary conservation analysis of *MYH3* amino acid sequence showed that these variants are highly conserved in the sarcomeric MYH genes expressed in the heart, and orthologous sequence alignment showed it to be conserved in chimp, mouse, rat, human and dog. Haplotype analysis showed that; the c.3574G>A (rs2285477) located at the tail domain, c.2952T>C (rs2285474) and c.2926-12A>G (rs2285473) located at the IQ domain showed strong correlation with ASD (p-value; GTA: 0.0003, ACG: 2.878e-5, ATG: 0.0293), the same LD block also showed association in 10,000 permutations [S3 Fig].

The amino acid substitution of hydrophobic alanine to hydrophilic theornine in p. Ala1752Thr and p. Ala1192Thr, places a hydrophobic side chain into a hydrophilic environment in the tail domain, which suggest that the mutant complex might be destabilized (ΔΔG of binding) relative to the wild-type complex. This action may pertubate the ability of the long coiled-coil tails of the individual myosin molecules to form thick filaments of the sarcomere. Thus further differing the ability of C-terminal tail domain’s ability for the heavy chains to self-associate and/or bind the myosin to its cargo [27]. A less stable thick filament could also affect the coupling between ATP hydrolysis and force generation by deficient transmission of energy from the motor domain to the sarcomeric structure [28].

Studies have also reported that mutations in the tail domain could lead towards disruption of either the head or the rod domain of embryonic myosin [29]. This result is similar to that conducted by Tajsharghi and colleagues [30], indicating that the tail domain is a functionally important residue for myosin heavy change (MHC) association, which binds thick filament in the cardiac muscle. A study by Ching and colleagues [31] identified I820N mutation that causes hydrophobic isoleucine to change to hydrophilic asparagine, thus predicting that the change may affect the binding of MHC to its regulatory light chain thus suggesting that these mutations may affect the normal formation of the myofibrils, which is in-hand with the findings of current study. However, histological analysis of the myocardium is substantially important to justify the said postulation.

The present study also identified c.2916A>G which is located at the late exonic position of exon 23 of the tail domain to affect splicing mechanism. *In silico* analysis suggested that this mutation will result in alterations of the exonic splicing enhancer (ESE) site, that would cause either skipping of the respective exon, activation of cryptic site or intron retention. Homology modelling of c. 3574G>A (p.Ala1192Thr) mutant against the wild type protein, showed some degree of difference with RMSD value of 5.4Å. However, these predictions were identified by the combined use of different algorithms where it is not yet possible to consistently predict the effects of nucleotide substitutions on splicing [32]. Therefore, *in vitro* analysis can be employed to further validate these predictions thus allowing a more accurate determination of the type of nucleotide changes which may affect the splicing mechanism.

*MYH3* plays important role in skeletal muscle development [33]. Other genes have additionally been reported to play important roles in skeletal muscle developments such as *MYH2*, *MYH7* and *MYH8* where myosin myopathies involving these genes have been widely reported. However, the phenotypes associated with these genes vary from one skeletal muscle gene to another, ranging from prenatal non-progressive arthrogryposis syndromes to adult-onset progressive muscle weakness [33]. Additionally, mutations in *MYH2* have been associated with dominant myopathy characterized by ophthalmoplegia, congenital joint contractures, and rimmed vacuoles in muscle fibers [34, 35]. Mutations in *MYH7* cause skeletal myopathies such as myosin storage myopathy and Laing early-onset distal myopathy [33, 34, 36, 37].

Although studies have reported the contributing factors of *MYH3* towards various syndromes including distal arthrogryposis type I, 2A (Freeman-Sheldon syndrome) and 2B (Sheldon-Hall syndrome), the association of *MYH3* towards heart abnormalities in humans has not been reported [29, 38]. The data presented in this study provided novel insights into the contribution of molecular genetics of *MYH3* towards the development of atrial septa.

Nevertheless, our study has some limitations, only *MYH3* gene was focussed, thus novel variants or other genomic loci beyond *MYH3*, was not identified. The identified variants were also detected among controls, therefore further study on the mendelian segregation among familial ASD patient for mutation in MYH3 is important. However, despite its limitations, targeted NGS is able to obtain high-quality data, within a hypothesis-driven approach, while remaining less expensive than its whole genome sequencing and whole exome sequencing counterparts. This methodology is appropriate for efficient and directed research, in elucidating molecular pathways of various diseases.

## Conclusion

In conclusion, this study proposes non-synonymous mutations in the tail domain of *MYH3* as a potential autosomal cause of ASD among non-syndromic patients. Hence, if a number of genes with contributions of similar magnitude as *MYH3* to the risk of ASD could be identified, these genes can be used as panel for diagnosis of ASD.

## Supporting information

S1 Fig Visualization of the LRPCR-NGS workflow. A) Schematic representation of *MYH3* long-range amplicons. B) Gel electrophoresis picture showing optimised amplicons; Lane M = 1 kb ladder, Lane A1 = Amplicon 1, Lane A2 = Amplicon 2, Lane 3 = Amplicon 3, Lane A4 = Amplicon 4, Lane–ve = Negative control; 0.7% agarose gel at 85V for 60 mins. C): Miseq reads aligned with *MYH3* loci of human genome assembly hg19 using BWA program and viewed using IGV; Red areas: reads from the plus DNA strands; blue area:reads from the minus strands.
(PDF)

S2 Fig ESE finder analysis of (A) wild-type and (B) mutated *MYH3* for c.2916A>G variant. The default threshold values for SF2/ASF (SRSF1), SC35 (SRSF2), SRp40 (SRSF5) and SRp55 (SRSF6) were 1.956, 2.383, 2.67 and 2.676 respectively. The width of each bar reflects the length of the motif, the placement of each bar along the X-axis represents the position of a motif along the DNA sequence, the height of the bar represents the numerical score on the Y-axis.
(PDF)

S3 Fig Permutation based analysis for haplotype block association for rs2285477, rs2285474 and rs2285473. The X-axis corresponds to chi-square value of permutated SNPs and the Y-axis corresponds to number of permutation. Permutation progress bar indicates the tallest bar with highest permutated χ2 at >900 permutation.
(PDF)
