# Supplementary material for: Mutations in the tail domain of MYH3 contributes to atrial septal defect
Source: PLoS One. 2020 Apr 21;15(4):e0230982. doi: 10.1371/journal.pone.0230982 (PMC7173802; doi:10.1371/journal.pone.0230982)
Supplement: S1 Fig — A) Schematic representation of MYH3 long-range amplicons. B) Gel electrophoresis picture showing optimised amplicons; Lane M = 1 kb ladder, Lane A1 = Amplicon 1, Lane A2 = Amplicon 2, Lane 3 = Amplicon 3, Lane A4 = Amplicon 4, Lane–ve = Negative control; 0.7% agarose gel at 85V for 60 mins. C): Miseq reads aligned with MYH3 loci of human genome assembly hg19 using BWA program and viewed using IGV; Red areas: reads from the plus DNA strands; blue area:reads from the minus strands. (PDF) [file pone.0230982.s001.pdf]

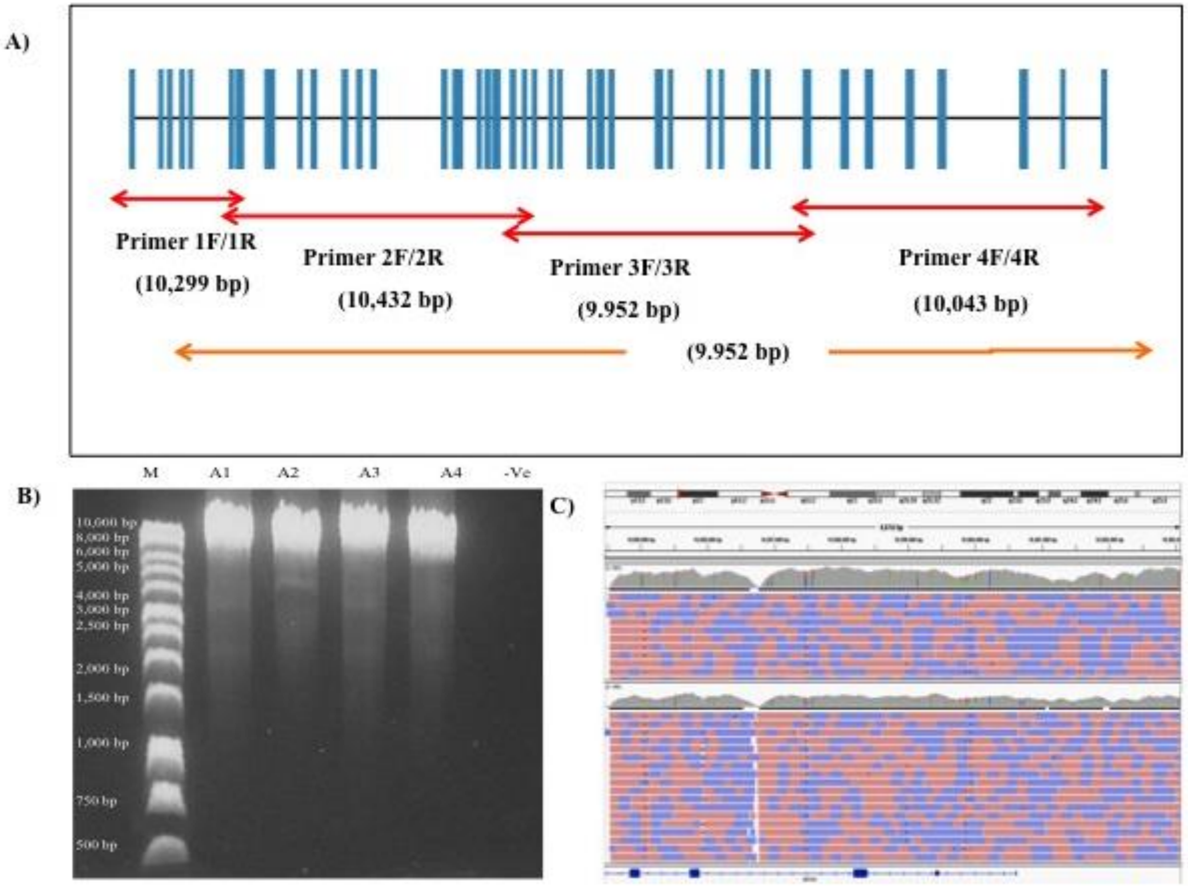

S1 Fig 1. Visualization of the LRPCR-NGS Workflow

A) Schematic representation of *MYH3* long-range amplicons. B) Gel electrophoresis picture showing optimised amplicons; Lane M=1 kb ladder, Lane A1=Amplicon 1, Lane A2= Amplicon 2, Lane 3=Amplicon 3, Lane A4=Amplicon 4, Lane –ve= Negative control; 0.7% agarose gel at 85V for 60 mins. C): Miseq reads aligned with *MYH3* loci of human genome assembly hg19 using BWA program and viewed using IGV; Red areas: reads from the plus DNA strands; blue area:reads from the minus strands.
