# Supplementary material for: Mutations in the tail domain of MYH3 contributes to atrial septal defect
Source: PLoS One. 2020 Apr 21;15(4):e0230982. doi: 10.1371/journal.pone.0230982 (PMC7173802; doi:10.1371/journal.pone.0230982)
Supplement: S2 Fig — ESE finder analysis of (A) wild-type and (B) mutated MYH3 for c.2916A>G variant. The default threshold values for SF2/ASF (SRSF1), SC35 (SRSF2), SRp40 (SRSF5) and SRp55 (SRSF6) were 1.956, 2.383, 2.67 and 2.676 respectively. The width of each bar reflects the length of the motif, the placement of each bar along the X-axis represents the position of a motif along the DNA sequence, the height of the bar represents the numerical score on the Y-axis. (PDF) [file pone.0230982.s002.pdf]

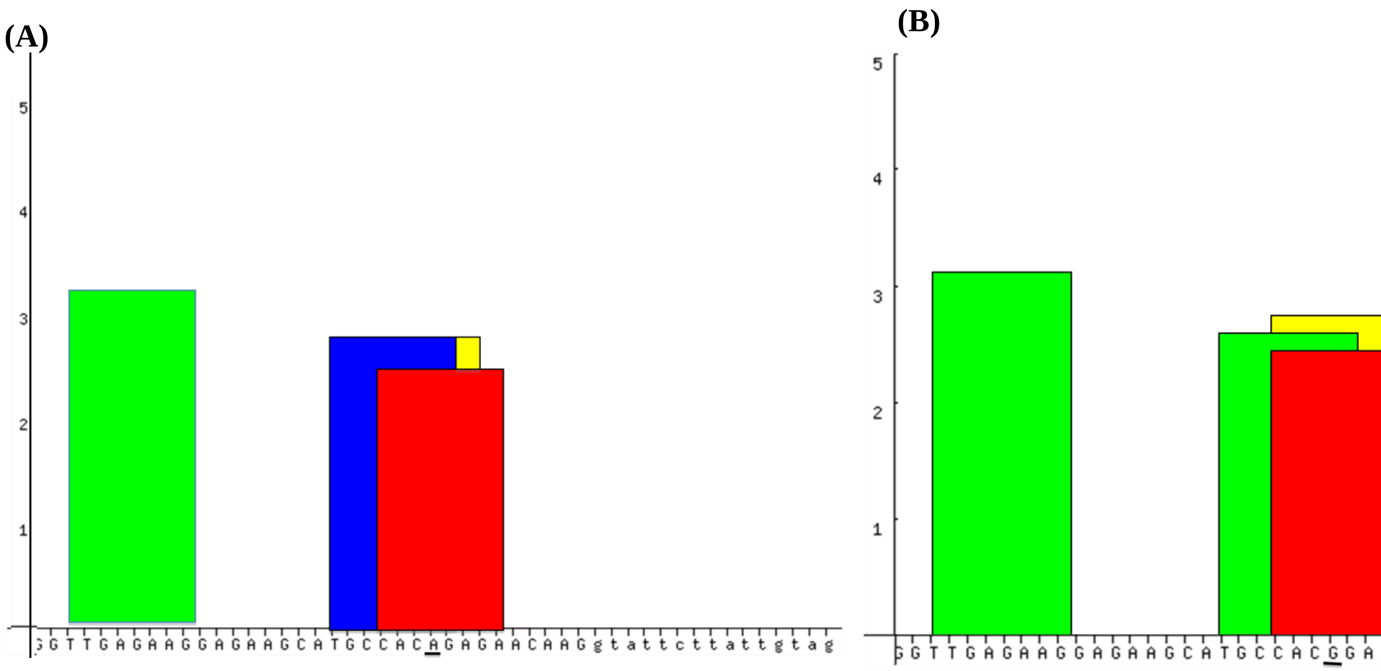


**S2 Fig 2. ESE finder analysis of (A) wild-type and (B) mutated *MYH3* for c.2916A>G variant.** The default threshold values for SF2/ASF (SRSF1), SC35 (SRSF2), SRp40 (SRSF5) and SRp55 (SRSF6) were 1.956, 2.383, 2.67 and 2.676 respectively. The width of each bar reflects the length of the motif, the placement of each bar along the X-axis represents the position of a motif along the DNA sequence, the height of the bar represents the numerical score on the Y-axis.
