# Supplementary material for: Mutations in the tail domain of MYH3 contributes to atrial septal defect
Source: PLoS One. 2020 Apr 21;15(4):e0230982. doi: 10.1371/journal.pone.0230982 (PMC7173802; doi:10.1371/journal.pone.0230982)
Supplement: S3 Fig — The X-axis corresponds to chi-square value of permutated SNPs and the Y-axis corresponds to number of permutation. Permutation progress bar indicates the tallest bar with highest permutated χ2 at >900 permutation. (PDF) [file pone.0230982.s003.pdf]

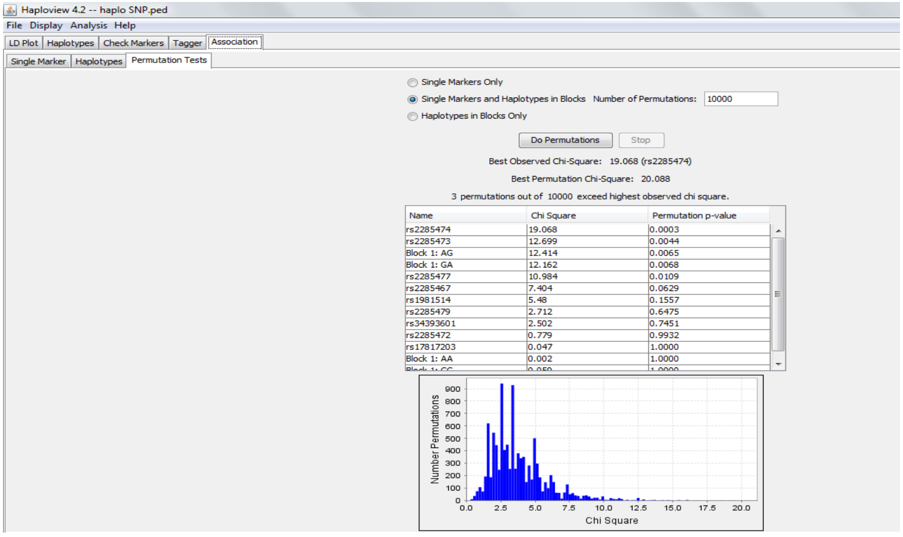

S3 Fig 3. Permutation based analysis for haplotype block association for rs2285477, rs2285474 and rs2285473.

The X-axis corresponds to chi-square value of permutated SNPs and the Y-axis corresponds to number of permutation. Permutation progress bar indicates the tallest bar with highest permutated χ^2 at >900 permutation
